# Preliminary Investigation on Mandarin Peel Extraction and Development of Functionalized Chitosan-Guar Gum Edible Films Using Response Surface Methodology (RSM)

**DOI:** 10.3390/foods15050803

**Published:** 2026-02-24

**Authors:** Miriam Arianna Boninsegna, Slaven Jurić, Amalia Piscopo, Marko Vuković, Zaixiang Lou, Luna Maslov Bandic

**Affiliations:** 1 Department of AGRARIA, University Mediterranea of Reggio Calabria, Via dell’Università 25, 89124 Reggio Calabria, Italy; miriam.boninsegna@unirc.it (M.A.B.); amalia.piscopo@unirc.it (A.P.); 2 Faculty of Agriculture, University of Zagreb, Svetošimunska 25, 10000 Zagreb, Croatia; sjuric@agr.hr (S.J.); mvukovic@agr.hr (M.V.); 3 State Key Laboratory of Food Science and Technology, Jiangnan University, Wuxi 214122, China; louzaixiang@126.com

**Keywords:** mandarin peel, ultrasound-assisted extraction, bioactive compounds, response surface methodology, chitosan-guar gum edible films

## Abstract

Every year worldwide, citrus processing generates large volumes of by-products, often wasted, although rich in bioactive compounds. In this study, mandarin peel (*Citrus reticulata*) was used as a source of functional compounds for the development of guar gum/chitosan functionalized edible films. The response surface methodology was used for both bioactive extraction and edible film formulation. For extraction, the optimization focused on extraction time, solvent composition (acetone/water ratio), and solvent/solid ratio, while for edible film, the guar gum/chitosan ratio, glycerol content, and mandarin peel extract concentration were selected as critical formulation variables. The predictive models exhibited high statistical significance (*p* < 0.05), adequate predictive ability, and good consistency of predicted and experimental values. The extraction optimization allowed significant results in total polyphenols (329.59 mg GAE/g), flavonoids (42.6 mg QE/g), and total carotenoids (1.53 mg/g) associated with significant antioxidant activity. Mandarin peel bioactive compounds integrated into composite edible film resulted in excellent functional properties in terms of swelling index (65.83%), water absorption (65.48%), weight loss (41.91%) and visual appearance (L* 89.30). These findings support formulating chitosan–guar gum films with mandarin peel bioactives, advancing biopolymer-based approaches toward next-generation sustainable packaging.

## 1. Introduction

The agri-food industry faces an urgent need to convert waste into value. Food processing industries generate significant amounts of by-products each year, posing serious environmental, logistical, and economic challenges [1]. Upcycling strategies are growing increasingly relevant in several production segments, among them cosmetics, pharmaceuticals, and livestock, highlighting the interdisciplinary relevance of agri-food by-products [2,3]. In the food sector, their conversion into high-value resources encourages the principles of the circular bioeconomy, reducing the ecological footprint of food industries and supporting the development of new, suitable food ingredients or eco-friendly packaging solutions [1,4,5].

Citrus by-products represent some of the most abundant in terms of volume, accounting for approximately 15 million tons annually [6]. Among these, mandarin (*Citrus reticulata*) by-products exhibit a valuable phytochemical composition, including flavonoids (hesperidin, eriocitrin, narirutin and naringin) [7], carotenoids (β cryptoxanthin, lutein, and zeaxanthin) [8], and essential oils (limonene, β myrcene, 3 carene, and α pinene) [9,10].

The efficiency and quality of bioactive compound recovery from food by-products depend on agronomic and technological factors [11,12,13,14]. Several authors have increasingly focused on “Green Chemistry” approaches, using eco-friendly solvents and non-thermal extraction techniques [15,16] combined with statistical approaches, such as the response surface methodology (RSM) to enhance extraction efficiency and reproducibility [17].

The recovery of bioactive compounds from citrus by-products and integration into edible coatings [18,19] to improve antioxidants and antimicrobial properties leads to extending the shelf-life of various food categories [20]. For instance, the application of *Citrus limetta* peel extract to enrich the carrageenan/alginate bio-nanocomposite film and *Citrus lemon* pomace extract in alginate-based coatings extended the shelf-life of meat and ready-to-eat fruit, respectively, compared to conventional storage conditions with an enhanced antioxidant and antimicrobial activity [21,22,23].

The formulation of active edible films, based on biopolymers, using bioactive compounds extracted from vegetable materials requires screening of the film-forming matrix, since its physical and chemical properties influence the overall performance of the final material, including stability and activity of release [24].

Polysaccharides, such as chitosan and guar gum, represent an efficient matrix, since they already exhibit good film-forming properties and positive interactions with embedded antioxidants and antimicrobial molecules derived from food by-products [25,26,27]. At the same time, both polymers are water sensitive, whose functional properties are closely influenced by polymer-water interactions [25,26].

Chitosan, a cationic polysaccharide derived from the deacetylation of chitin, possesses well-recognized characteristics, including structural integrity, strength, gas and light barrier and excellent antimicrobial properties against both Gram-positive and Gram-negative bacteria.

Guar gum, a highly hydrophilic galactomannan, is characterized by strong water affinity and high viscosity, which enhance film flexibility and homogeneity but also limit barrier and mechanical performance when used alone, thus motivating its combination with other biopolymers (e.g., carrageenan, chitosan) to improve the structural and functional cohesion of final composite edible films [28]. Bioactive compounds derived from agri-food by-products with different polarities, such as phenolic and carotenoid fractions, have been shown to modulate intermolecular interactions and water-related physical properties in polysaccharide-based matrices [27]. In this context, chitosan–guar gum blends represent a suitable model system to explore the effects of citrus by-product extracts, such as mandarin peel extract, on polymer-water interactions and related physical properties [27,28].

This study implemented a dual-stage RSM strategy integrating bioactive extraction from mandarin peel (*Citrus reticulata*) and edible film formulation as a material and process design framework. The first stage focused on optimizing the extraction recovery of bioactive compounds (polyphenols, flavonoids, and carotenoids) by using solvents (acetone/water mixture) and non-thermal techniques (ultrasound-assisted extraction). The second stage optimized the formulation of edible composite films based on guar gum and chitosan by adjusting the ratios of biopolymers, mandarin peel extract and plasticizer, to elucidate the role of the mandarin extract in shaping the physical properties of the polymeric matrix. Considering the hydrocolloidal nature of guar gum and chitosan, swelling index, total water solubility and film weight loss were selected as target responses.

The study’s purpose was to provide a preliminary process-oriented framework, designed with a low-impact laboratory-scale approach, encompassing the extraction of bioactive compounds, the use of renewable edible biopolymers, and the formulation of composite films through controlled processing strategies. Within this framework, the mandarin peel extract is incorporated into the formulation as a controllable variable within the experimental design, moving beyond the fixed-dose or limited-concentration approaches commonly adopted in previous studies [23,24,25,28]. This methodological choice enables a process-oriented evaluation of extract–matrix interactions, contributing to the rational design of environmentally friendly edible film systems.

## 2. Materials and Methods

### 2.1. Plant Material

The mandarin fruits (*Citrus reticulata*) were obtained by the local producer Novallis (Croatia). After receiving them in the laboratories of the Faculty of Agriculture, University of Zagreb, mandarin peel was manually removed, lyophilized, ground into a fine powder using a laboratory mill, and then sieved through a stainless-steel sieve with a mesh diameter of 450 µm to standardize the upper particle size limit (<450 μm). The powders thus obtained were stored at −20 °C and then used for the extraction process.

### 2.2. Experimental Procedure to Optimize the Mandarin Peel Extract

The mandarin (*Citrus reticulata*) peel bioactive compounds extraction process was optimized using a Box-Behnken experimental design within the response surface methodology (RSM), implemented using Design-Expert^®^ software (Version 7.0.0, State-Ease Inc., Minneapolis, MN, USA). A three-factor Box-Behnken design (BBD) was used to study the effect of three independent variables: (i) raw material concentration (0.10–0.30% *w*/*v*), (ii) extraction time (1–15 min), and (iii) acetone: water mixture (60–100% *v*/*v*). The model lack-of-fit was tested using replicated center points to determine pure error. The ultrasound amplitude was set to 50% (UP200St ultrasonic processor—Sonotrode S26d14 (Heilscher Ultrasonics GmbH, Teltow, Germany) with a frequency of 25.8 kHzTotal phenolic content (TPC), total flavonoid content (TFC), total carotenoid content (TCC), and antioxidant activity (ABTS and DPPH assays) were used as responses to optimize the extraction process. The experimental design is reported in Table S1 (Supplementary data).

#### 2.2.1. Total Phenolic Content, Total Flavonoid Content, Total Carotenoid Content, and Antioxidant Activity (ABTS and DPPH Assays) of Mandarin Peel Extract

Total phenolic compounds (TPC) of mandarin peel extract (MPE) were evaluated using the modified method proposed by Singleton [29]. Briefly, 100 µL extract was mixed with 7.9 mL deionized water, 0.5 mL Folin-Ciocalteau (diluted 1:2 (*v*/*v*) with distilled water) and 1.5 mL of the aqueous solution of Na_2_CO_3_ (20%, *w*/*v*). Then, the mixture was vortexed and kept in the dark room at an environmental temperature of 25 °C. After 2 h, the absorbance was detected at 765 nm using a spectrophotometer (Shimadzu, Kyoto, Japan) and the results were expressed as mg gallic acid equivalent (GAE) g^−1^ of mandarin peel. Total flavonoids (TFC) compounds were evaluated using the method proposed by Ivanova et al. [30]. The reaction mixture was prepared as follows: 1 mL of sample, 6.4 mL of deionized water and 0.3 mL of 0.1% (*w*/*v*) NaNO_2_ were placed in a test tube, and after 5 min, 0.3 mL of 0.05% (*w*/*v*) AlCl_3_ was mixed in. Finally, after 6 min, the reaction mixture was completed by adding 2 mL of NaOH 1 M and vortexing. The absorbance was reached at 360 nm using a spectrophotometer (Shimadzu, Kyoto, Japan), and the results were expressed as mg quercetin equivalent (QE) g^−1^ of mandarin peel. The total carotenoid content (TCC) was determined using the protocol proposed by Bandić et al. [31]. The extracts obtained from the extractions were placed in a quartz cuvette, and the absorbance was recorded at 450 nm using a spectrophotometer (Shimadzu, Kyoto, Japan). The results were expressed as mg β-carotene g^−1^ of mandarin peel using a calibration curve of the β-carotene standard prepared in different concentrations ranging from 1 to 30 mg L^−1^.

The antioxidant capacity of extracts originating from mandarin peel was assessed using 2,2-diphenyl-1-picrylhydrazyl (DPPH) and 2,2′-azino-bis (3-ethylbenzothiazoline-6-sulphonic acid) (ABTS) radicals, according to the methods described by Jurić et al. [32]. After extraction, the final volume of each sample was adjusted to 100 mL to standardize extract concentration according to the fixed solid-to-solvent ratio (% *w*/*v*) (Table S1).

To perform the DPPH assay, 0.1 mL of extract was mixed with 3.9 mL of a methanolic DPPH solution, left to react for 30 min (in the dark), and subsequent spectrophotometric determination of discoloration was carried out at 517 nm against the methanol blank. Instead, the ABTS assay was executed by mixing 40 μL of extract in 4 mL of a radical ABTS solution prepared and stabilized in accordance with Boninsegna et al. [33]. After 6 min of incubation, the absorbance of the prepared reaction mixture was read at 734 nm against a blank consisting of water. A calibration curve was built using Trolox ((±)-6-hydroxy-2,5,7,8-tetramethylchroman-2-carboxylic acid), and the results are expressed in mmol of Trolox equivalents per mL of sample.

#### 2.2.2. The HPLC Analysis

The obtained extracts were evaporated to dryness and reconstituted in 4 mL of water. An aliquot of 0.6 mL of the sample was mixed with 0.6 mL of a methanol/DMSO (1:1, *v*/*v*) solution in Eppendorf tubes. The mixture was sonicated for 15 min at 50 °C and then centrifuged at 5000 rpm for 15 min at 4 °C. Before the HPLC analysis, samples were filtered through 0.45 μm PTFE membranes. The HPLC analyses were performed using an Agilent Infinity II system (Agilent, Palo Alto, CA, USA) equipped with a diode array detector. The separation of flavonoids was carried out on an Agilent Poroshell 120 SB-C18 column (150 × 4.6 mm, 4 µm; Agilent, Palo Alto, CA, USA) according to the method described by Maslov Bandić et al. [34].

### 2.3. Experimental Design to Optimize the Formulation of Guar Gum-Chitosan Films

**Figure A1:**
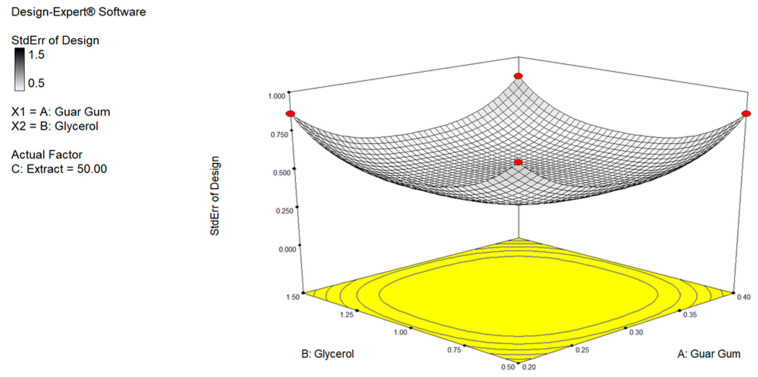
3D surface and corresponding contour plot of the standard error of the RSM experimental design as a function of guar gum/chitosan ratio (A) and glycerol concentration (B), with MPE concentration fixed at 50. The distribution of design points, including replicated center points, highlights the structure and coverage of the experimental design reported in Table 1.

The formulation of the edible film was optimized by implementing the response surface methodology (RSM) approach, using a Box–Behnken design (BBD) with three independent variables: (i) guar gum (GG)/Chitosan (CH) ratio (0.13–29); (ii) glycerol concentration (0.50–1.5% *w*/*v*); and (iii) mandarin peel extract (MPE) concentration (0–50% *v*/*v*). A total of 17 experimental tests were generated to study the interaction effects of these components on the functional performance (swelling index, total water absorption, and film weight loss) of the resulting films (Table 1 and Figure A1).

The design matrix and the experimental data were analyzed using Design-Expert^®^ Software (Version 7.0.0, State-Ease Inc., Minneapolis, MN, USA) to fit a quadratic model, assess statistical significance (via ANOVA), determine the interaction between the factors, and predict optimal formulation conditions. Three-dimensional surface plots were also generated to visualize the relationships between variables and responses.

#### 2.3.1. Formulation of Composite Guar Gum/Chitosan Films Loaded with Mandarin Peel Extract

After the extraction procedure, the best extract in terms of bioactive compounds and antioxidant activity was filtered through Whatman No. 4 filter paper, and the solvent was removed by a rotary evaporator at a fixed temperature of 50 °C (BUCHI Rotavapor R-300) until 5% of the initial solution. This was further used to prepare enriched edible films.

The solutions of guar gum and chitosan were made as described by Bhan et al. [35]. Briefly, the guar gum solution was prepared by dissolving an appropriate quantity of guar gum powder (0.10–0.20% *w*/*v*) in 100 mL of distilled water (30 °C) under constant stirring until complete dissolution (4 h). Then the solution was degassed in an ultrasound bath for 15 min.

The chitosan solution was prepared by dissolving a suitable amount of chitosan low molecular weight powder (0.70–0.80% *w*/*v*) in 100 mL of distilled water, containing 1% (*v*/*v*) of acetic acid. The solution was kept under constant stirring for 4 h until complete dissolution at room temperature (25 ± 5 °C). The suspension was then filtered with a Büchner funnel and a Whatman No. 4 paper filter. Finally, the solution was degassed in an ultrasound bath for 15 min.

Finally, the guar gum and chitosan were blended in different ratios (from 0.13 to 0.29) according to the experimental design shown in Table 1. Then MPE (0–50% *v*/*v*) was added to the formulation, and glycerol (0.5–1.5% *w*/*v*) was incorporated as a plasticizer to improve the film’s flexibility. Solutions thus formulated were mixed under constant stirring for 15 min using a rotavapor to avoid the formation of bubbles.

For film casting, 50 mL of each solution was placed in Petri dishes (90 mm diameter) and air-dried for 36 h at 50 °C.

#### 2.3.2. Physical Properties of Composite Guar Gum/Chitosan Films Loaded with MPE: Color Measurement, Swelling Index, Total Water Absorption, and Film Weight Loss

Color measurements were conducted by using a colorimeter (ColorTec PCM; ColorTec Associates Inc., Clinton, NJ, USA) and according to the CIE L*a*b* and CIE L*C*h◦ systems (Commission International d’eclairage). Since measured films are partly transparent, a white calibration plate was placed above them, as reported in Etxabide et al. [36]. After colorimeter calibration, three measurements were collected on each film from which average values were obtained. The obtained values were observed as relative, like the difference between measured films or a white plate. In a three-dimensional uniform space, the L* variable is the vertical coordinate that describes lightness, and a* and variables are horizontal ones, which, when negative, indicate an intensity of green and blue color (respectively), and if positive intensity of red and yellow color (respectively) [37]. Toward to the international criterion CIELAB, when hue angle (h°) is 0°, it assigns to the semi axis +a* (redness); when 90°, it assigns to the semi axis +b* (yellowness); when 180°, it assigns to the semi axis −a* (greenness); and when 270°, it assigns to the semi axis −b* (blueness).

**Figure 1:**
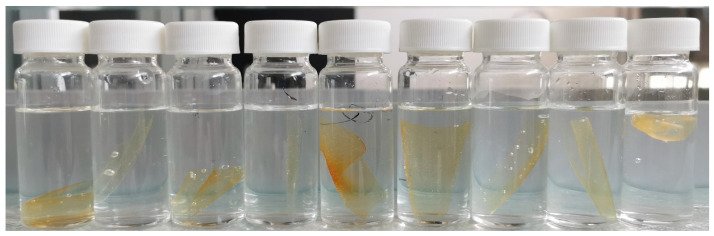
Films immersed in deionized water after 24 h.

The swelling index (SI), total water absorption (TWA), and film weight loss (FWL) were investigated by methods proposed by Shang et al. [38] with some modifications. Briefly, the hydrogel films (1 cm × 3 cm) were dried at 60 °C until they reached a consistent weight to obtain the dry film weight (W_0_). The hydrogel films were immersed in 20 mL of deionized water at 25 °C for 24 h (Figure 1).

After one day, the water on the surface of the film was carefully eliminated with filter paper and then weighed (W_s_). Finally, the hydrogel films were subjected to drying in a controlled environment at 60 °C until they reached a consistent weight (W_f_).

Swelling index (SI), total water absorption (TWA), and film weight loss/solubility (FWL) were calculated from the following equations:

(1) $$SI=\frac{W_{s}-W_{0}}{W_{0}}\times 100$$

(2) $$TWA=\frac{W_{s}-W_{f}}{W_{s}}\times 100$$

(3) $$FWL=\frac{W_{0}-W_{f}}{W_{0}}\times 100$$

## 3. Results and Discussion

### 3.1. Model Fitting to Optimize the Recovery of Bioactive Compounds of Mandarin Peel

Five responses relating to the total content of polyphenols (TPC), flavonoids (TFC), and carotenoids (TCC) and to their antioxidant activity, assessed by ABTS and DPPH radical scavenging assays, were used to make second-order polynomial models for optimizing the extraction process of the principal bioactive compounds present in mandarin peel. The adequacy and predictive performance of the fitted models were verified through analysis of variance (ANOVA). Table 2 lists the results of the ANOVA with the most relevant statistical validation parameters, confirming that the models were reliable (*p* < 0.0001). The R^2^, adjusted R^2^, and predicted R^2^ values were also high and mutually consistent for TPC, TFC and DPPH, as was the Adeq Precision, which measures the signal-to-noise ratio, exceeding the threshold value of 4 in all models, confirming strong predictive capacity and validating the fit of the model for design space exploration and optimization [39,40].

However, the models related to ABTS and TCC exhibited a statistically significant lack of fit, indicating a discrepancy between experimental and predicted values. The observations were consistent with the findings of several authors, whereby model non-fit was attributable to a variety of causes, including the contamination of the theoretical model [41], the inadequate representation of the real system by second-order polynomial equations [42], or the presence of latent variables not considered in the experimental design [43].

The second-order regression equations obtained by the Box-Behnken design (Table 3) were examined to evaluate the contributions. The analysis of the regression coefficients confirmed the ANOVA observations (Table 2), highlighting that the significant models corresponded to the TPC, TFC, and DPPH responses for both linear terms and quadratic terms, particularly A^2^ and C^2^. Significant interaction terms, such as AB and BC, also indicated synergistic or antagonistic effects between specific factors [44,45]. The TCC and ABTS models, on the other hand, revealed lower predictive capacity due to the presence of insignificant regression terms or less consistency between the observed and predicted data.

In this study, the adequacy of the quadratic models developed was further confirmed by analyzing the normal residual plots for each response (Figure S3). The clustering points exhibited roughly linear distributions around the diagonal line, suggesting that the error normality hypothesis can be satisfied [46].

These results support the overall predictive accuracy of the RSM models used in the multivariate extraction process optimization, also confirming the validity of the polynomial equations shown in Table 2 in accurately describing the experimental system’s behavior [47].

However, while the residual distribution analysis allowed us to check the model’s conformity with regression assumptions, as the normality of errors, it was also essential to integrate global statistical parameters (R^2^, adj-R^2^, and pred-R^2^) and the significance of the model terms to guarantee the model’s predictive ability, as suggested by Chen et al. [42], Montgomery [43], and Khuri and Mukhopadhyay [48].

#### 3.1.1. Effect of Extraction Conditions on TPC, TFC and TFC

**Figure 2:**
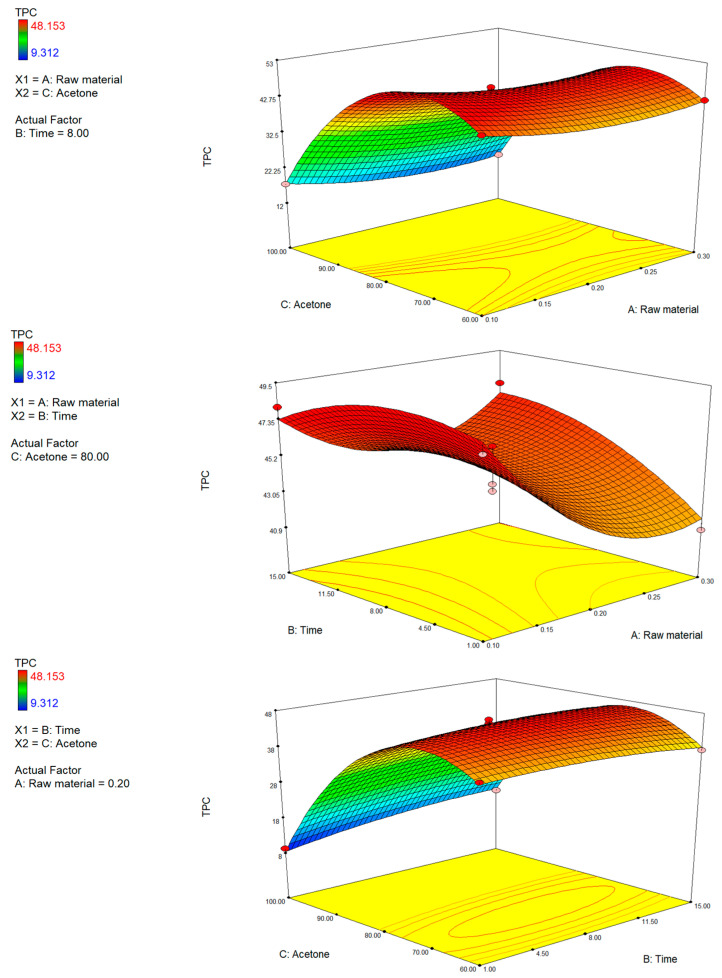
3D plots of total phenolic content (TPC) showing the interactive effects of (A) raw material and extraction time, (B) raw material and acetone concentration, and (C) extraction time and acetone concentration.

**Figure 3:**
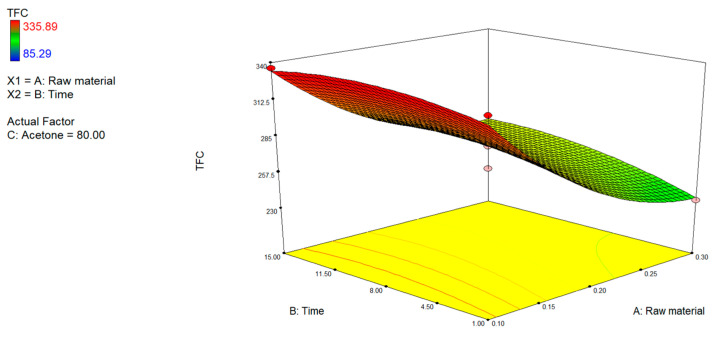

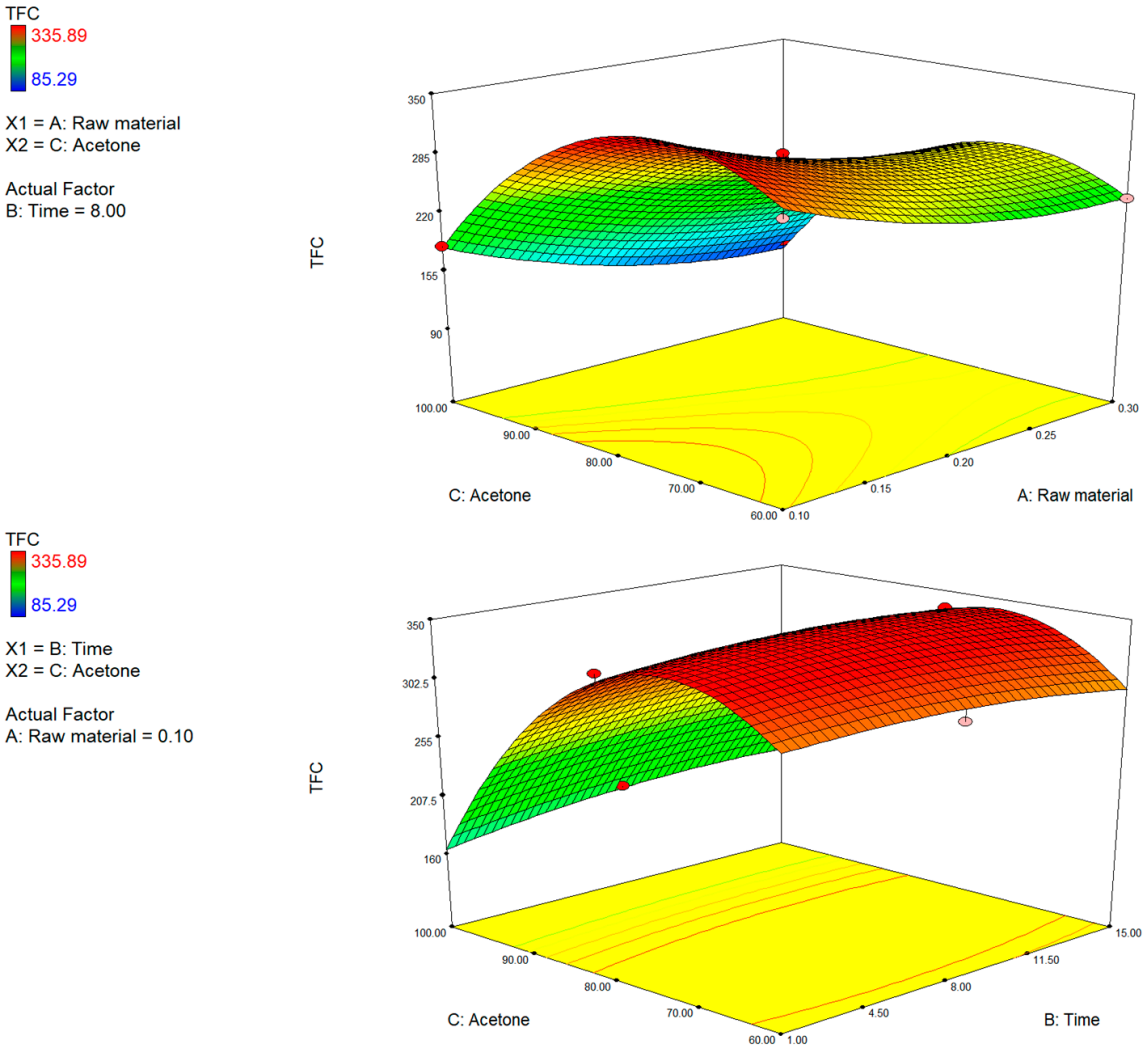
3D plots of total flavonoid content (TFC) showing the interactive effects of (A) raw material and extraction time, (B) raw material and acetone concentration, and (C) extraction time and acetone concentration.

**Figure 4:**
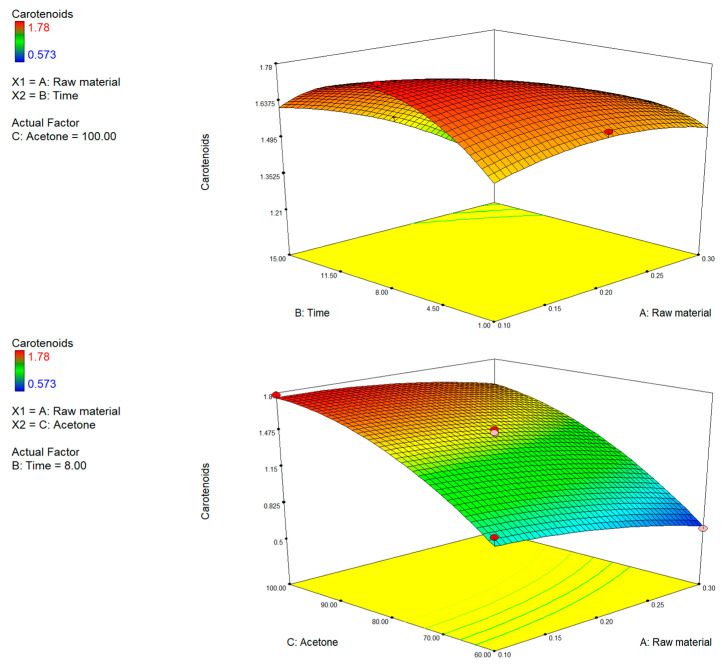

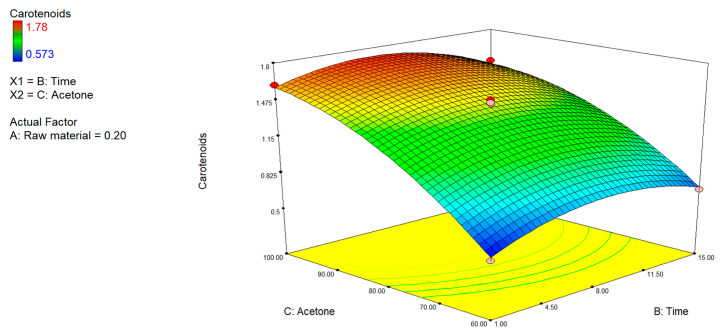
3D plots of total carotenoid content (TCC) showing the interactive effects of (A) raw material and extraction time, (B) raw material and acetone concentration, and (C) extraction time and acetone concentration.

The comparative analysis of the three-dimensional response surfaces relating to TPC (Figure 2), TFC (Figure 3), and TCC (Figure 4) showed a comparable trend to the extraction process variables investigated. The most influential variable on the recovery process of bioactive compounds was the solvent polarity. An increase in the percentage of acetone in the extraction mixture correlated with an improvement in extraction efficiency. The data revealed agreement with earlier studies on the extraction of bioactive compounds from citrus by-products, in which it was pointed out that these compounds exhibit a varied chemical nature and were characterized by variable lipophilicity, making them more soluble in apolar or semipolar organic environments, such as those containing acetone [49].

Conversely, both high concentrations of the raw material and extending the extraction time beyond optimal thresholds led to a significant decline in yields, likely due to solvent saturation, mass-transfer inefficiencies, or thermal and oxidative degradation [50,51]. This effect is particularly marked in the yield of carotenoids, compounds that are photosensitive, thermolabile, and prone to oxidation [52,53,54].

The analysis of the response surface graphs suggests that the optimal operative conditions that were shared for the three groups of phytochemical extraction were levels of acetone > 70–80% (*v*:*v*), extraction times of 7–9 min, and a low concentration of the raw material (<0.2%).

#### 3.1.2. Effect of Extraction Parameters on Antioxidant Activity

**Figure 5:**
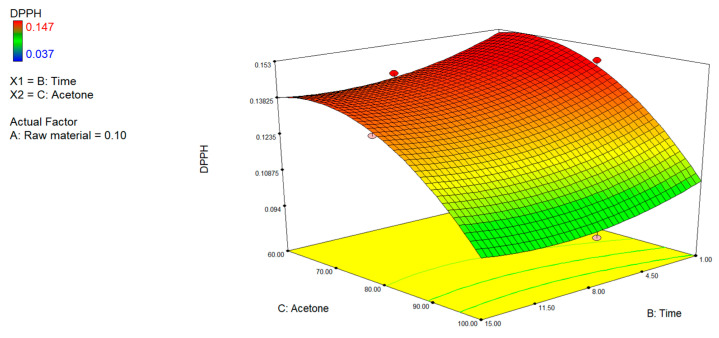

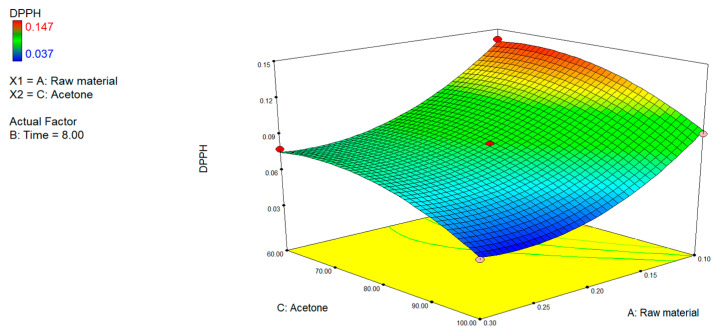
3D plots of total antioxidant activity (DPPH assay) showing the interactive effects of raw material (A), extraction time (B), and acetone concentration (C).

**Figure 6:**
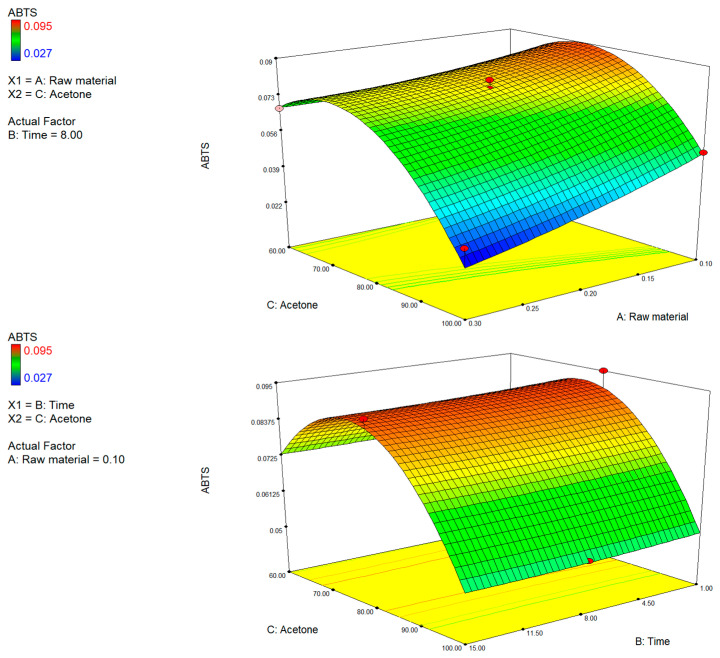
3D plots of total antioxidant activity (ABTS assay) showing the interactive effects of raw material (A), extraction time (B), and acetone concentration (C).

The free radical-scavenging activity of the extracts, evaluated using the ABTS and DPPH assays, showed different trends with respect to the process variables tested (Figure 5 and Figure 6). The DPPH model particularly demonstrated greater consistency and predictive capacity compared to the ABTS model, as also confirmed by the respective R^2^, Adeq precision, and lack of fit values (Table 2).

This was highly dependent on the chemical nature of the bioactive compounds recovered, the composition of the solvent system, and the type of radical involved in the assay [55,56]. DPPH (2,2-diphenyl-1-picrylhydrazyl) is mainly based on hydrogen atom transfer and, to a lesser extent, electron transfer. Therefore, it is selective to compounds able to donate the hydrogen effectively, such as flavonoids with a catechol structure (e.g., quercetin, luteolin) or carotenoids with well-exposed conjugated double bonds (β-carotene, lutein) [57,58,59]. The ABTS (2,2′-azinobis-3-ethylbenzothiazoline-6-sulfonic acid), on the other hand, reacts by electron transfer and potentially responds to a larger range of molecules, including phenolic acids (ferulic, caffeic), although it is also more susceptible to variability due to the nature of the solvent and the polarity of the compounds [57,58,59,60].

Overall, both assays showed greater antioxidant activity with higher acetone content (>80%), low raw material concentration (<0.2%), and moderate extraction times (6–9 min), confirming that bioactive compounds with enhanced antioxidant activity were recovered with low-polarity solvent systems and under conditions that hindered phenomena of degradation or oversaturation of the system.

#### 3.1.3. Validation of Predicted Optimum and Extract Characterization

The response surface method was employed to determine the extraction conditions to maximize key parameters (total polyphenol content, flavonoids, carotenoids, and antioxidant activity). Optimal conditions obtained through RSM modeling were raw material 0.1%, time of 8.97 min, and 80.06% (*v*/*v*) of acetone (Figure S4).

Table 4 reports in detail the chemical characterization of the extract obtained with operating conditions and theoretical predictions with deviations below 15%, confirming the valuable bioactive properties of mandarin peel and the predictive reliability of the models developed to maximize their recovery.

The quantification of bioactive compounds (Table 4) indicated that UAE, when combined synergistically with a suitable solvent system, extraction time, and solid-to-liquid ratio, markedly improved the recovery of total phenolic content (TPC) and total flavonoid content (TFC) compared to conventional extraction methods [12]. In fact, the traditional techniques, such as maceration and Soxhlet extraction, were reported to yield TPC values ranging from approximately 1 to 28 mg GAE g^−1^ and TFC values of about 3–4 mg CE g^−1^ [12,61], with a hesperidin content varying between 1.00 and 1.34 mg g^−1^, despite requiring prolonged extraction times and higher process severity.

This finding was linked to the mechanistic features of ultrasound-assisted extraction. Unlike Soxhlet extraction and maceration, which rely on diffusion-driven kinetics and prolonged thermal exposure, ultrasound-assisted extraction enhanced mass transfer through cavitation-induced microbubble formation and cell wall disruption, promoting the weakening of hydrogen bonding and non-covalent interactions within the plant matrix and enabling the controlled desorption and solubilization of phenolic compounds, flavonoids, and moderately lipophilic carotenoids [12,61]. Moreover, unlike microwave-assisted extraction, which primarily relies on dipole rotation and ionic conduction and may induce localized overheating, the ultrasound-driven process ensured a more homogeneous energy distribution, thereby limiting oxidative reactions and thermally induced degradation pathways of labile bioactive molecules [12].

Therefore, the RSM-optimized ultrasound-assisted extraction as adopted in this study enabled the recovery of a total phenolic content of 329.6 mg GAE g^−1^, together with substantially higher levels of total flavonoids and carotenoids, within an extraction time of less than 9 min. In addition, the high abundance of key flavonoids, particularly hesperidin (59.59 mg g^−1^) and narirutin (2.35 mg g^−1^), recognized markers of *Citrus reticulata* peels and closely associated with antioxidant and protective properties [62,63,64], confirmed the effectiveness of the optimized extraction strategy adopted, and was consistent with previous reports on citrus matrices processed using sustainable assisted extraction technologies [65,66,67].

Overall, this integrated process design enabled a more efficient and controlled recovery of both polar and moderately lipophilic bioactive compounds from mandarin peel, defining the added value of the proposed method over traditional extraction techniques in terms of extraction efficiency, process sustainability, and preservation of compound integrity.

### 3.2. Model Fitting to Optimize the Formulation of Guar Gum-Chitosan Films

The results obtained for the analysis of variance (ANOVA) for the Response Surface Quadratic Model (Table 5) and the derived regression equations suggested that the quadratic models accurately represented the relationship between the independent variables (guar gum, glycerol, and MPE extract) and the technological responses of the films. The F values for the swelling index (SI = 10.13), total water absorption (TWA = 11.72), and film weight loss (FWL = 25.82) models were statistically significant (*p* < 0.05), minimizing the possibility that the results were due to experimental noise (0.01–0.30%). The high Adeq precision values also confirmed the excellent signal-to-noise ratio, an essential requirement for ensuring the reliability of the model in describing the experimental space [68]. However, there were discrepancies for SI between Pred-R^2^ and Adj-R^2^, suggesting a potential margin for improvement in the predictability of the model. This was in line with what had already been observed in studies conducted on bio-polymer-based edible films [68,69,70,71], where difficulties were encountered in modeling the swelling properties, such as the one proposed in this study, due to the variability of hydrocolloid systems [69,70,71].

Although the swelling index (SI) model exhibited a low Pred-R^2^ value (0.0477), the model remained statistically significant (*p* = 0.0030), showed a high Adj-R^2^ (0.8369), Adeq precision above the recommended threshold (>4), and a non-significant lack of fit (*p* = 0.0592). This behavior reflects the intrinsic complexity of swelling phenomena in hydrocolloid-based systems rather than a deficiency in the experimental design.

Swelling behavior in polysaccharide films is governed by highly non-linear polymer–water interactions, microstructural heterogeneity, chain relaxation, and threshold effects, which are often difficult to accurately describe using second-order polynomial equations, particularly within limited Box–Behnken experimental designs.

The quadratic equations (Table 6) represented a predictive tool, providing an interpretative key to the biopolymer-based film-forming matrix’s molecular performance.

The significance of the quadratic terms (A^2^, B^2^, and C^2^) indicated the polymeric and cross-linked nature of the system, whereby small variations in composition resulted in amplified responses in swelling and weight loss properties, consistent with the findings of Mohammadi et al. [71] and Zhang et al. [72], who suggested that non-linear interactions between polysaccharides and plasticizers were crucial in modulating the functionality of nanocomposite films. Similarly, significant interactions (AB and AC) indicated that the synergistic nature between the components of the edible film determined an important effect in substantially modulating properties that are essential for edible films [72,73,74].

Against this background, consistency between Adeq precision values (>4) and the equations’ capacity to describe experimental responses highlights the predictive reliability of the models presented in this study. This approach is part of a well-established line of research that values response surface methodology as a key methodology for reducing experimental complexity and increasing control in formulation processes [68,71].

The standardized internal error distribution, reported in the normal residual plots (Figure S5), supported the good fit of the polynomial models developed in this study, displaying a distribution of points along the theoretical diagonal without systematic deviations for swelling index (Figure S5a), total water absorption (Figure S5b), and film weight loss (Figure S5c). This trend followed the principle of normality of error distribution, an essential requirement for the validation of the analysis of variance and the RSM model, following Mohammadi et al. [71] in the development of antimicrobial nanocomposite films. Specifically, TWA and FWL showed similar patterns with the points linearly along the diagonal indicating correct coverage of the design space [74]. Likewise, the SI trend, although showing a slight deviation at the lowest portion of the scale (studentized residuals lower than -1) associated with the low experimental values recorded, supported the overall reliability of the model as they exhibited a general absence of accumulations or recurring patterns and uniform color distribution.

The analysis of ANOVA (Table 5), regression equations (Table 6) and residual plots (Figure S5) support the developed models. These findings were consistent with the emphasis placed by Zhang and colleagues [72] on the importance of normal plot analysis to identify deviations of modeling hypotheses for complex RSM studies, particularly in the presence of multiple simultaneous responses in multiphase film-forming systems.

#### 3.2.1. Effect of Formulation Parameters on the Physical Properties of Composite Films Guar Gum/Chitosan

Analysis of the experimental data relating to the 17 composite edible film formulations (Table 7) revealed considerable variability in the films’ performance responses (Figures S6–S8) as a function of the different guar gum/chitosan ratio, glycerol and MPE, showing SI values ranging from 56.22% to 513.17%, TWA from 64.22% to 91.70%, and FWL from 37.26% to 94.11%. The high sensitivity of the functional properties of the edible film to formulation factors correspond results of the ANOVA analysis (Table 5), in which the quadratic terms B^2^ (glycerol), C^2^ (MPE extract) and A^2^ (guar gum) were statistically significant for the modeled responses. The data also confirmed what was observed in previous studies on the gelation kinetics of chitosan, where a strong sensitivity to experimental conditions linked to specific concentrations of polymeric and gelling components in multiphase film-forming systems was found [75].

**Figure 7:**
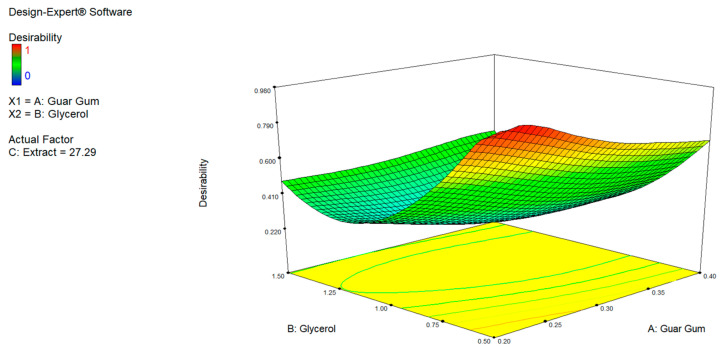
3D Desirability graph showing the combined effect of guar gum/chitosan ratio and glycerol concentration on overall desirability, with MPE fixed at 27.29%. Higher desirability values identify the optimal formulation region.

The three-dimensional desirability graph (Figure 7) confirmed the observations in Table 7, highlighting an optimal area corresponding to a guar gum/chitosan ratio between 0.13 and 0.20 and glycerol between 0.50% and 0.75%, with a fixed concentration of MPE at 27.29%. In this region, the highest overall desirability values (up to 0.98) were achieved, suggesting that the equilibrium between hydrocolloids and plasticizer maximized the functional performance of the proposed composite edible film.

#### 3.2.2. Validation of the Predicted Model to Optimize Composite Edible Films Guar Gum/Chitosan Loaded with MPE

The optimization strategy based on the simultaneous minimization of SI (swelling index), TWA (total water absorption), and FWL (film weight loss) was designed to enhance the physical stability of edible film under real storage conditions, supporting the functionality of the polymer system in concrete food packaging scenarios [75]. These responses were chosen based on biopolymers used being inherently water-sensitive and their performance depending on water-polymer interactions, making them a critical design parameter for regulating water sensitivity in extract-loaded edible films [27,28].

Table 8 shows the predicted results compared to the experimental values in the optimal formulation conditions, which included a 0.14 guar gum/chitosan ratio, 0.50% glycerol, and 27.29% extract MPE. The predicted values closely followed the experimental values, with deviations < 11% for each response (10.78% SI, 3.39% TWA and 3.71% FWL), and the total desirability of the model of 0.977, supporting the predictive validity and robustness of the developed mathematical model [71,72].

In real food applications, minimizing water-related parameters plays a key role in preventing structural degradation and preserving the functional properties of edible films over time, such as those proposed in this study. The low swelling avoids the film’s expansion upon exposure to water, thus preserving its properties, whilst the low water absorption and low weight loss also provide essential support for the long-term structural integrity and barrier performance [76]. The literature highlights the relevance of water-related film properties in the context of foods characterized by high water activity, such as fresh-cut vegetables, fresh cheeses, and meats, owing to their sensitivity to chemical–physical and microbiological alterations [77].

The edible film also exhibited excellent color characteristics, L* = 90.62, which conferred a degree of transparence favorable to food application, as well as a* and b* values of –4.03 and 22.58, respectively, indicating a slight yellow-green predominance typical of coatings containing flavonoids and carotenoids [78,79]. The hue angle (h*) value of 100.11 was also in the yellow-green quadrant, consistent with the phytochemical composition of the extract, while the moderate chroma (C*) value of 22.11 gave a natural and visually neutral appearance to the film, consistent with reports for other bioactive systems [80,81]. The observed color characteristics were associated with the phytochemical composition of the MPE, indicating the presence of bioactive components, such as carotenoids, flavonoids and other phenolic molecules, which have been reported in previous studies to exhibit photoprotective and antioxidant functions toward photosensitive substrates, including lipids, pigments, and vitamins, thereby suggesting promising perspectives for the functional valorization of the MPE-loaded edible composite films formulated in the present study [80,82].

The enhanced performance demonstrated by the optimized formulation of the guar gum chitosan film enriched with MPE was due to the balancing of the biopolymer combination, plasticizer and MPE concentration. These mechanisms were also supported by current scientific literature [1,25,27,83], which already reported that chitosan, because of their cationic structure rich in protonable amino groups, resulted in the formation of cross-linked networks through hydrogen bonds and electrostatic interactions [25], while guar gum also contributed to the film-forming matrix’s stickiness and cohesion, modulating the diffusion of water and glycerol [84]. Furthermore, the incorporation of antioxidant extracts derived from by-products, such as mandarin peel extract, rich in bioactive compounds (including hesperidin and narirutin), led to an improvement in key parameters, such as water absorption and solubility, enhancing its overall performance [74,80,85]. Zhang et al. [72], by optimizing carboxymethyl chitosan and gelatin-based films with RSM, observed that intermediate levels of the factors contributed to a functional synergy between hydrocolloid components and plasticizing agents, which positively impacted the flexibility/dimensional stability equilibrium. In addition, Rahman et al. [86], comparing a pure chitosan edible film and a guar–chitosan cross-linked film, found that the composite films exhibited a better mechanical strength of 39 ± 1.15 MPa and reduced vapor permeability compared to pure chitosan, as indeed Bhowmik et al. [87] confirmed that the addition of functional compounds, such as chito-oligosaccharides or phenolic acids in chitosan-based active packaging, resulted in a considerable effect on cross-linking, favoring the formation of more resistant cross-linked networks, which also enhanced mechanical properties and UV protection.

## 4. Conclusions

This study demonstrated the mandarin peel as a promising source of bioactive compounds within a preliminary, process-oriented framework for the design of sustainable edible films, linking extraction optimization and material formulation.

The results support a rational, design-driven approach based on the integration of dual optimization strategies through response surface methodology, which enabled the maximization of the extraction efficiency of compounds exhibiting high antioxidant activity. Furthermore, the integration of the mandarin peel extract into a guar gum/chitosan matrix resulted in edible composite films characterized by high performance in terms of reduced swelling index, water absorption, and weight loss, as well as desirable color characteristics. Future research should investigate scalability, storage stability, and real application scenarios of these films, including the assessment of mechanical, functional, and antimicrobial performance, in order to validate their practical applicability in food systems and to further develop the process-oriented framework proposed in the present preliminary study.

## Figures and Tables

**Table 1:** Experimental procedure to optimize the formulation of guar gum/chitosan edible film.

Samples	F1 GG/CH Ratio	F2 Glycerol (%)	F3 MPE (%)
1	0.20	1.00	33.33
2	0.29	1.50	33.33
3	0.20	1.00	33.33
4	0.29	1.00	50.00
5	0.20	0.50	0.00
6	0.13	1.00	50.00
7	0.20	1.50	50.00
8	0.20	1.00	33.33
9	0.20	1.00	33.33
10	0.13	1.00	0.00
11	0.13	1.50	33.33
12	0.13	0.50	33.33
13	0.29	0.50	33.33
14	0.20	1.00	33.33
15	0.20	0.50	50.00
16	0.20	1.50	0.00
17	0.29	1.00	0.00

**Table 2:** ANOVA for the quadratic models of carotenoids, TFC, TPC, DPPH, and ABTS as influenced by raw material concentration (A), extraction time (B), and acetone concentration (C).

Response	Source	Sum of Squares	df	Mean Square	F-Value	*p*-Value Prob > F	Other
Carotenoids	Model	2.07	9	0.23	67.37	<0.0001	Std. Dev. = 0.058
	A-Raw material	0.14	1	0.14	39.61	0.0004	Mean = 1.26
	B-Time	2.101 × 10^−4^	1	2.101 × 10^−4^	0.062	0.8112	C.V.% = 4.65
	C-Acetone	1.55	1	1.55	454.55	<0.0001	PRESS = 0.37
	AB	0.043	1	0.043	12.49	0.0095	R2 = 0.9886
	AC	4.556 × 10^−3^	1	4.556 × 10^−3^	1.33	0.2858	Adj R2 = 0.9739
	BC	0.011	1	0.011	3.29	0.1125	Pred R2 = 0.8246
	A2	0.014	1	0.014	4.22	0.0790	Adeq Precision = 25.791
	B2	0.15	1	0.15	44.18	0.0003	
	C2	0.13	1	0.13	38.54	0.0004	
	Residual	0.024	7	3.413 × 10^−3^			
	Lack of Fit	0.023	3	7.616 × 10^−3^	29.18	0.0035	
	Pure Error	1.044 × 10^−3^	4	2.610 × 10^−4^			
	Cor Total	2.09	16				
TFC	Model	3058.32	9	339.81	240.78	<0.0001	Std. Dev. = 1.19
	A-Raw material	29.13	1	29.13	20.64	0.0027	Mean = 37.02
	B-Time	6.95	1	6.95	4.93	0.0619	C.V.% = 3.21
	C-Acetone	1631.32	1	1631.32	1155.89	<0.0001	PRESS = 68.61
	AB	10.54	1	10.54	7.47	0.0292	R2 = 0.9968
	AC	0.94	1	0.94	0.67	0.4416	Adj R2 = 0.9926
	BC	14.47	1	14.47	10.25	0.0150	Pred R2 = 0.9776
	A2	31.95	1	31.95	22.64	0.0021	Adeq Precision = 44.179
	B2	7.01	1	7.01	4.96	0.0611	
	C2	1331.88	1	1331.88	943.72	<0.0001	
	Residual	9.88	7	1.41			
	Lack of Fit	3.68	3	1.23	0.79	0.5582	
	Pure Error	6.20	4	1.55			
	Cor Total	3068.20	16				
TPC	Model	88,803.28	9	9867.03	81.70	<0.0001	Std. Dev. = 10.99
	A-Raw material	12,541.32	1	12,541.32	103.84	<0.0001	Mean = 240.34
	B-Time	555.94	1	555.94	4.60	0.0691	C.V.% = 4.57
	C-Acetone	36,753.03	1	36,753.03	304.31	<0.0001	PRESS = 7208.32
	AB	116.10	1	116.10	0.96	0.3595	R2 = 0.9906
	AC	89.87	1	89.87	0.74	0.4169	Adj R2 = 0.9784
	BC	259.53	1	259.53	2.15	0.1861	Pred R2 = 0.9196
	A2	1814.16	1	1814.16	15.02	0.0061	Adeq Precision = 28.596
	B2	244.34	1	244.34	2.02	0.1979	
	C2	36,852.99	1	36,852.99	305.14	<0.0001	
	Residual	845.41	7	120.77			
	Lack of Fit	407.78	3	135.93	1.24	0.4049	
	Pure Error	437.63	4	109.41			
	Cor Total	89,648.70	16				
DPPH	Model	0.014	9	1.510 × 10^−3^	141.82	<0.0001	Std. Dev. = 3.263 × 10^−3^
	A-Raw material	7.381 × 10^−3^	1	7.381 × 10^−3^	693.06	<0.0001	Mean = 0.087
	B-Time	1.800 × 10^−5^	1	1.800 × 10^−5^	1.69	0.2348	C.V.% = 3.73
	C-Acetone	3.160 × 10^−3^	1	3.16 × 10^−3^	296.73	<0.0001	PRESS = 1.008 × 10^−3^
	AB	5.625 × 10^−5^	1	5.625 × 10^−5^	5.28	0.0551	R2 = 0.9945
	AC	9.000 × 10^−6^	1	9.000 × 10^−6^	0.85	0.3885	Adj R2 = 0.9875
	BC	6.250 × 10^−6^	1	6.25 × 10^−6^	0.59	0.4687	Pred R2 = 0.9263
	A2	1.883 × 10^−3^	1	1.883 × 10^−3^	176.85	<0.0001	Adeq Precision = 42.400
	B2	1.466 × 10^−4^	1	1.466 × 10^−4^	13.76	0.0076	
	C2	1.058 × 10^−3^	1	1.058 × 10^−3^	99.32	<0.0001	
	Residual	7.455 × 10^−5^	7	1.065 × 10^−5^			
	Lack of Fit	6.175 × 10^−5^	3	2.058 × 10^−5^	6.43	0.0520	
	Pure Error	1.280 × 10^−5^	4	3.200 × 10^−6^			
	Cor Total	0.014	16				
ABTS	Model	6.049 × 10^−3^	9	6.721 × 10^−4^	14.41	0.0010	Std. Dev. = 6.830 × 10^−3^
	A-Raw material	5.780 × 10^−4^	1	5.780 × 10^−4^	12.39	0.0097	Mean = 0.067
	B-Time	4.050 × 10^−5^	1	4.050 × 10^−5^	0.87	0.3824	C.V.% = 10.24
	C-Acetone	2.312 × 10^−3^	1	2.312 × 10^−3^	49.57	0.0002	PRESS = 4.877 × 10^−3^
	AB	3.600 × 10^−5^	1	3.600 × 10^−5^	0.77	0.4088	R2 = 0.9488
	AC	1.210 × 10^−4^	1	1.210 × 10^−4^	2.59	0.1513	Adj R2 = 0.8829
	BC	0.000	1	0.000	0.000	1.0000	Pred R2 = 0.2350
	A2	1.684 × 10^−5^	1	1.684 × 10^−5^	0.36	0.5668	Adeq Precision = 12.839
	B2	1.053 × 10^−6^	1	1.053 × 10^−6^	0.023	0.8848	
	C2	2.957 × 10^−3^	1	2.957 × 10^−3^	63.39	<0.0001	
	Residual	3.265 × 10^−4^	7	4.664 × 10^−5^			
	Lack of Fit	3.025 × 10^−4^	3	1.008 × 10^−4^	16.81	0.0099	
	Pure Error	2.400 × 10^−5^	4	6.000 × 10^−6^			
	Cor Total	6.376 × 10^−3^	16				

**Table 3:** Final equations in terms of coded and actual factors (Bioactive compounds).

	Final Equations in Terms of Coded Factors	Final Equations in Terms of Actual Factors
Carotenoids	+1.46 − 0.13 × A − 5.125 × 10^−3^ × B + 0.44 × C − 0.10 × A × B + 0.034 × A × C − 0.053 × B × C − 0.059 × A^2^ − 0.19 × B^2^ − 0.18 × C^2^	−3.55711 + 0.87000 × Raw material + 0.12085 × Time + 0.092372 × Acetone − 0.14750 × Raw material × Time + 0.016875 × Raw material × Acetone − 3.78571 × 10^−4^ × Time × Acetone − 5.85000 × Raw material^2^ − 3.86224 × 10^−3^ × Time^2^ − 4.41875 × 10^−4^ × Acetone^2^
TFC	+44.70 − 1.91 × A + 0.93 × B − 14.28 × C + 1.62 × A × B − 0.48 × A × C + 1.90 × B × C + 2.75 × A^2^ − 1.29 × B^2^ − 17.79 × C^2^	−162.12870 − 128.44979 × Raw material − 0.99617 × Time + 6.33995 × Acetone + 2.31929 × Raw material × Time − 0.24225 × Raw material × Acetone + 0.013584 × Time × Acetone + 275.48250 × Raw material^2^ − 0.026325 × Time^2^ − 0.044464 × Acetone^2^
TPC	+278.18 − 39.59 × A + 8.34 × B − 67.78 × C + 5.39 × A × B − 4.74 × A × C + 8.05 × B × C + 20.76 × A^2^ − 7.62 × B^2^ − 93.56 × C^2^	−793.62921 − 1098.19893 × Raw material − 2.46382 × Time + 34.04681 × Acetone + 7.69643 × Raw material × Time − 2.37000 × Raw material × Acetone + 0.057536 × Time × Acetone + 2075.72500 × Raw material^2^ − 0.15546 × Time^2^ − 0.23389 × Acetone^2^
DPPH	+0.082 − 0.030 × A − 1.500 × 10^−3^ × B − 0.020 × C + 3.750 × 10^−3^ × A × B + 1.500 × 10^−3^ × A × C + 1.250 × 10^−3^ × B × C + 0.021 × A^2^ + 5.900 × 10^−3^ × B^2^ − 0.016 × C^2^	+0.089156 − 1.25261 × Raw material − 3.9265 × 10^−3^ × Time + 5.1248 × 10^−3^ × Acetone + 5.357 × 10^−3^ × Raw material × Time + 7.500 × 10^−4^ × Raw material × Acetone + 8.9286 × 10^−6^ × Time × Acetone + 2.11500 × Raw material^2^ + 1.20408 × 10^−4^ × Time^2^ − 3.96250 × 10^−5^ × Acetone^2^
ABTS	+0.078 − 8.500 × 10^−3^ × A + 2.250 × 10^−3^ × B − 0.017 × C + 3.000 × 10^−3^ × A × B − 5.500 × 10^−3^ × A × C + 0.000 × B × C + 2.000 × 10^−3^ × A^2^ + 5.000 × 10^−4^ × B^2^ − 0.026 × C^2^	− 0.29206 + 0.020714 × Raw material − 6.98980 × 10^−4^ × Time + 0.010300 × Acetone + 4.28571 × 10^−3^ × Raw material × Time − 2.75000 × 10^−3^ × Raw material × Acetone + 0.000000 × Time × Acetone + 0.20000 × Raw material^2^ + 1.02041 × 10^−5^ × Time^2^ − 6.62500 × 10^−5^ × Acetone^2^

**Table 4:** Predicted and experimental responses to optimal conditions in terms of raw material 0.1%, time (8.97 min), and acetone (80.06% *v*/*v*) for maximized values for responses.

	TCC (mg β-Carotene g^−1^)	TFC (mg QE g^−1^)	TPC (mg GAE g^−1^)	DPPH (mmol TE g^−1^)	ABTS (mmol TE g^−1^)	Desirability
Predicted	1.539	49.191	338.535	0.133	0.088	0.912
Experimental	1.533	42.598	329.59	0.121	0.083	
Predicted vs. experimental (%)	0.39	13.40	2.64	9.02	5.68	

**Table 5:** ANOVA Response for the quadratic models of swelling index (SI), total water absorption (TWA), and film weight loss (FWL).

Response	Source	Sum of Squares	df	Mean Square	F-Value	*p*-Value Prob > F	Other
SI	Model	4283.36	9	475.93	10.13	0.0030	Std. Dev. = 6.86
	A-Guar Gum	138.83	1	138.83	2.95	0.1294	Mean = 55.50
	B-Glycerol	84.55	1	84.55	1.80	0.2217	C.V.% = 12.35
	C-Extract	34.89	1	34.89	0.74	0.4175	PRESS = 4392.42
	AB	309.21	1	309.21	6.58	0.0373	R^2^ = 0.9287
	AC	15.53	1	15.53	0.33	0.5834	Adj R^2^ = 0.8369
	BC	24.87	1	24.87	0.53	0.4906	Pred R^2^ = 0.0477
	A^2^	613.86	1	613.86	13.06	0.0086	Adeq Precision = 8.739
	B^2^	1314.37	1	1314.37	27.96	0.0011	
	C^2^	1374.54	1	1374.54	29.24	0.0010	
	Residual	329.03	7	47.00			
	Lack of Fit	268.63	3	89.54	5.93	0.0592	
	Pure Error	60.40	4	15.10			
	Cor Total	4612.39	16				
TWA	Model	1122.03	9	124.67	11.72	0.0019	Std. Dev. = 3.26
	A-Guar Gum	3.70	1	3.70	0.35	0.5739	Mean = 81.36
	B-Glycerol	292.84	1	292.84	27.52	0.0012	C.V.% = 4.01
	C-Extract	1.349 × 10^−3^	1	1.349 × 10^−3^	1.268 × 10^−4^	0.9913	PRESS = 847.92
	AB	53.24	1	53.24	5.00	0.0604	R^2^ = 0.9377
	AC	51.42	1	51.42	4.83	0.0639	Adj R^2^ = 0.8577
	BC	3.01	1	3.01	0.28	0.6112	Pred R^2^ = 0.2913
	A^2^	6.96	1	6.96	0.65	0.4452	Adeq Precision = 10.745
	B^2^	693.65	1	693.65	65.18	<0.0001	
	C^2^	1.43	1	1.43	0.13	0.7248	
	Residual	74.49	7	10.64			
	Lack of Fit	50.67	3	16.89	2.84	0.1699	
	Pure Error	23.82	4	5.96			
	Cor Total	1196.52	16				
FWL	Model	2611.10	9	290.12	25.82	0.0001	Std. Dev. = 3.35
	A-Guar Gum	19.91	1	19.91	1.77	0.2248	Mean = 56.37
	B-Glycerol	582.71	1	582.71	51.86	0.0002	C.V.% = 5.95
	C-Extract	10.02	1	10.02	0.89	0.3764	PRESS = 492.73
	AB	4.54	1	4.54	0.40	0.5453	R^2^ = 0.9708
	AC	230.11	1	230.11	20.48	0.0027	Adj R^2^ = 0.9332
	BC	0.35	1	0.35	0.031	0.8653	Pred R^2^ = 0.8168
	A^2^	446.01	1	446.01	39.70	0.0004	Adeq Precision = 17.258
	B^2^	179.14	1	179.14	15.94	0.0052	
	C^2^	1130.52	1	1130.52	100.62	<0.0001	
	Residual	78.65	7	11.24			
	Lack of Fit	25.62	3	8.54	0.64	0.6261	
	Pure Error	53.03	4	13.26			
	Cor Total	2689.75	16				

**Table 6:** Final equations in terms of coded and actual factors (Swelling Index, Total Water Absorption, and Film Weight Loss).

	*Final Equations in Terms of Coded Factors*	*Final Equations in Terms of Actual Factors*
SI	+78.00 + 4.17 × A + 3.25 × B − 2.09 × C − 8.79 × A × B + 1.97 × A × C − 2.49 × B × C − 12.07 × A^2^ − 17.67 × B^2^ − 18.07 × C^2^	−188.15046 + 922.26245 × Guar Gum + 205.58734 × Glycerol + 0.66246 × Extract − 175.84419 × Guar Gum × Glycerol + 0.39411 × Guar Gum × Extract − 0.099739 × Glycerol × Extract − 1207.44280 × Guar Gum^2^ − 70.67261 × Glycerol^2^ − 7.22720 × 10^−3^ × Extract^2^
TWA	+88.28 + 0.68 × A + 6.05 × B + 0.013 × C − 3.65 × A × B − 3.59 × A × C − 0.87 × B × C − 1.29 × A^2^ − 12.84 × B^2^ − 0.58 × C^2^	−23.75339 + 192.77610 × Guar Gum + 138.40722 × Glycerol + 0.27341 × Extract − 72.96752 × Guar Gum × Glycerol − 0.71711 × Guar Gum × Extract − 0.034709 × Glycerol × Extract − 128.58454 × Guar Gum^2^ − 51.34054 × Glycerol^2^ − 2.33112 × 10^−4^ × Extract^2^
FWL	+46.89 − 1.58 × A + 8.53 × B + 1.12 × C + 1.07 × A × B − 7.58 × A × C − 0.29 × B × C + 10.29 × A^2^ − 6.52 × B^2^ + 16.39 × C^2^	+ 99.40210 − 578.75795 × Guar Gum + 63.44946 × Glycerol − 0.16617 × Extract + 21.30359 × Guar Gum × Glycerol − 1.51692 × Guar Gum × Extract − 0.011799 × Glycerol × Extract + 1029.20507 × Guar Gum^2^ − 26.09074 × Glycerol^2^ + 6.55436 × 10^−3^ × Extract^2^

**Table 7:** Composition of the 17 composite edible film formulations and corresponding measured responses, including swelling index (SI), total water absorption (TWA), and film water solubility (FWL).

Samples	F1 GG/CH Ratio	F2 Glycerol (%)	F3 Extract (%)	SI (%)	TWA (%)	FWL (%)
1	0.20	1.00	33.33	292.22	86.16	45.71
2	0.29	1.50	33.33	75.26	76.80	59.33
3	0.20	1.00	33.33	323.04	88.61	51.81
4	0.29	1.00	50.00	149.71	86.80	67.03
5	0.20	0.50	0.00	74.57	70.76	48.96
6	0.13	1.00	50.00	62.41	90.02	83.79
7	0.20	1.50	50.00	58.29	77.22	63.95
8	0.20	1.00	33.33	406.73	89.21	45.33
9	0.20	1.00	33.33	302.83	85.71	42.43
10	0.13	1.00	0.00	65.85	78.85	64.93
11	0.13	1.50	33.33	159.39	85.32	61.92
12	0.13	0.50	33.33	56.22	64.22	44.11
13	0.29	0.50	33.33	111.20	70.29	37.26
14	0.20	1.00	33.33	513.17	91.70	49.14
15	0.20	0.50	50.00	57.94	68.55	50.34
16	0.20	1.50	0.00	112.00	82.90	63.75
17	0.29	1.00	0.00	114.34	89.97	78.51

**Table 8:** Predicted and experimental responses to optimal conditions in terms of SI (swelling index), TWA (total water absorption), and FWL (film weight loss) for maximized values for responses.

	SI	TWA	FWL	Desirability
Predicted	58.73	63.33	40.41	0.977
Experimental	65.83	65.48	41.91	
Predicted vs. experimental (%)	10.78	3.39	3.71	

## Data Availability

The original contributions presented in the study are included in the article. Further inquiries can be directed to the corresponding author.

## Supplementary Materials

The following supporting information can be downloaded at https://www.mdpi.com/article/10.3390/foods15050803/s1, Figure S1: Lyophilized mandarin peels (particle size < 450 µm); Figure S2: Mandarin peel suspension before (a) and after the filtration (b) and subjection to evaporation (c); Figure S3: Normal plots of residuals for Carotenoids (a), Flavonoids (b), Polyphenols (c) DPPH (d), and ABTS (e) response. The near-linear distribution of points indicates normality of residuals; Figure S4: Response surface plot of overall desirability showing the combined effects of raw material concentration (A) and extraction time (B) at a fixed acetone concentration (80%). Maximum desirability (0.92) was achieved at 0.1% raw material, 8.97 min extraction time, and 80.06% (*v*/*v*) acetone; Figure S5: Normal plots of residuals for swelling index (a), total water absorption (b), and film weight loss (c) response. The near-linear distribution of points indicates normality of residuals; Figure S6: 3D plots of swelling index (SI) showing the interactive effects of guar gum/chitosan ratio (A), Glycerol (B), and MPE extract (C); Figure S7: 3D plots of total water absorption (TWA) showing the interactive effects of guar gum/chitosan ratio (A), Glycerol (B), and MPE extract (C); Figure S8: 3D plots of film weight loss (FWL) showing the interactive effects of guar gum/chitosan ratio (A), Glycerol (B), and MPE extract (C); Table S1: Experimental design to optimize the mandarin peel extraction.

## Abbreviations

The following abbreviations are used in this manuscript:

RSM	Response Surface Methodology
BBD	Box–Behnken Design
MPE	Mandarin Peel Extract
TPC	Total Phenolic Content
TFC	Total Flavonoid Content
TCC	Total Carotenoid Content
DPPH	2,2-Diphenyl-1-Picrylhydrazyl
ABTS	2,2′-Azino-Bis (3-Ethylbenzothiazoline-6-Sulphonic Acid)
GAE	Gallic Acid Equivalent
QE	Quercetin Equivalent
TE	Trolox Equivalent
GG	Guar Gum
CH	Chitosan
SI	Swelling Index
TWA	Total Water Absorption
FWL	Film Weight Loss
