# Supplementary material for: Preliminary Investigation on Mandarin Peel Extraction and Development of Functionalized Chitosan-Guar Gum Edible Films Using Response Surface Methodology (RSM)
Source: Foods. 2026 Feb 24;15(5):803. doi: 10.3390/foods15050803 (PMC12985080; doi:10.3390/foods15050803)
Supplement: Supplementary file 1 [file foods-15-00803-s001.zip › foods-4125419-supplementary.pdf]

Supplementary Data

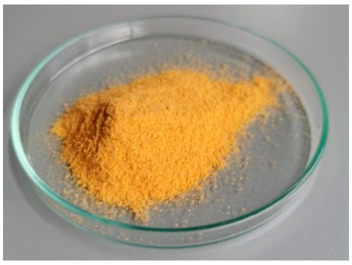

Figure S1. Lyophilized mandarin peels (particle size <450 μm)

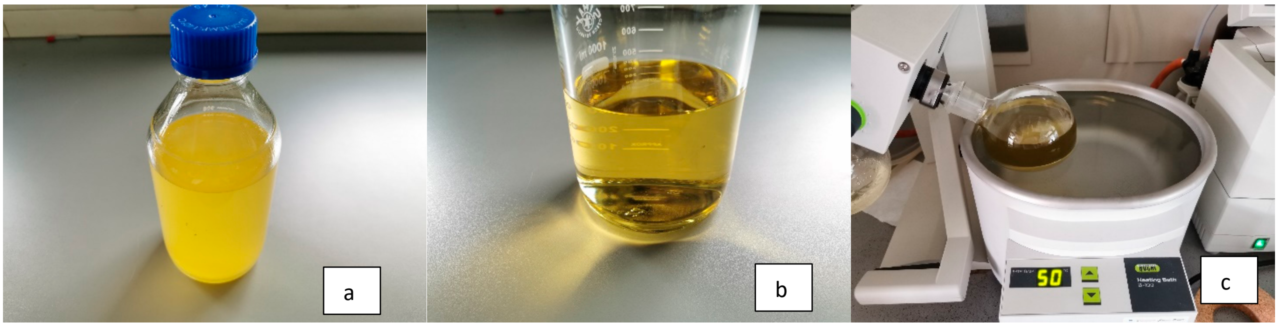

Figure S2. Mandarin peel suspension before (a) and after the filtration (b) and subjection to evaporation (c).

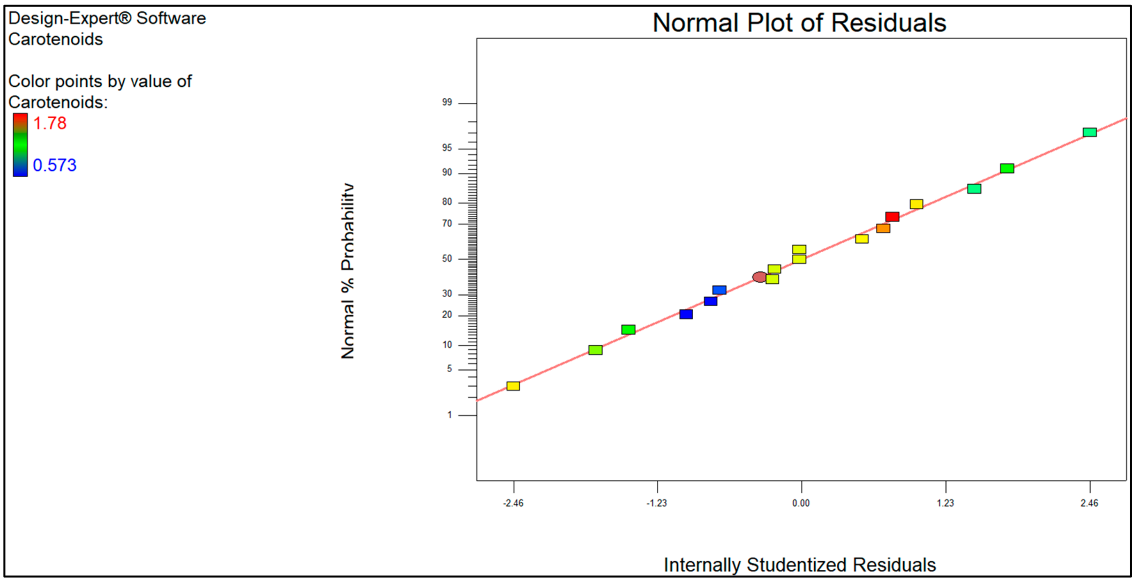

(a)

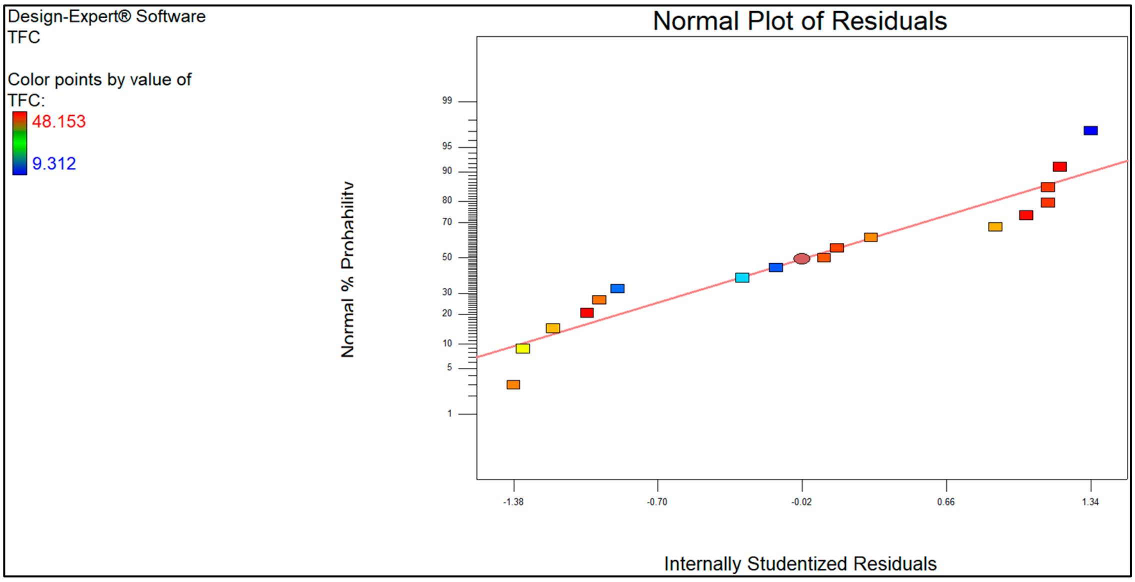
Design-Expert® Software
TFC
Color points by value of
TFC:
48.153
9.312
Normal Plot of Residuals
Normal % Probability
99
95
90
80
70
50
30
20
10
5
1
-1.38
-0.70
-0.02
0.66
1.34
Internally Studentized Residuals

(b)

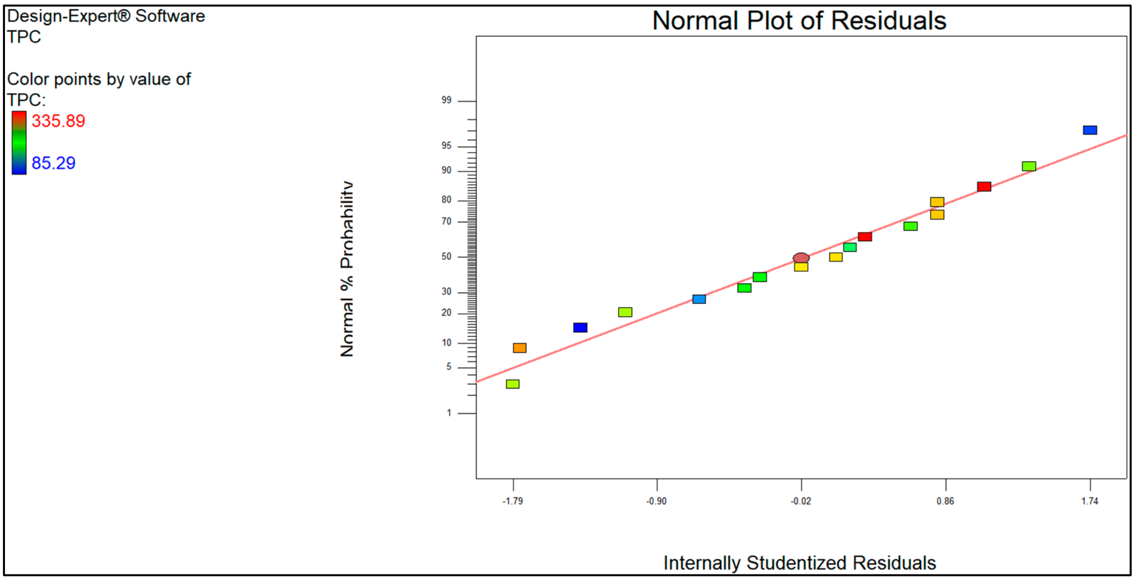
Design-Expert® Software
TPC
Color points by value of
TPC:
335.89
85.29
Normal Plot of Residuals
Normal % Probability
99
95
90
80
70
50
30
20
10
5
1
-1.79
-0.90
-0.02
0.86
1.74
Internally Studentized Residuals

(c)

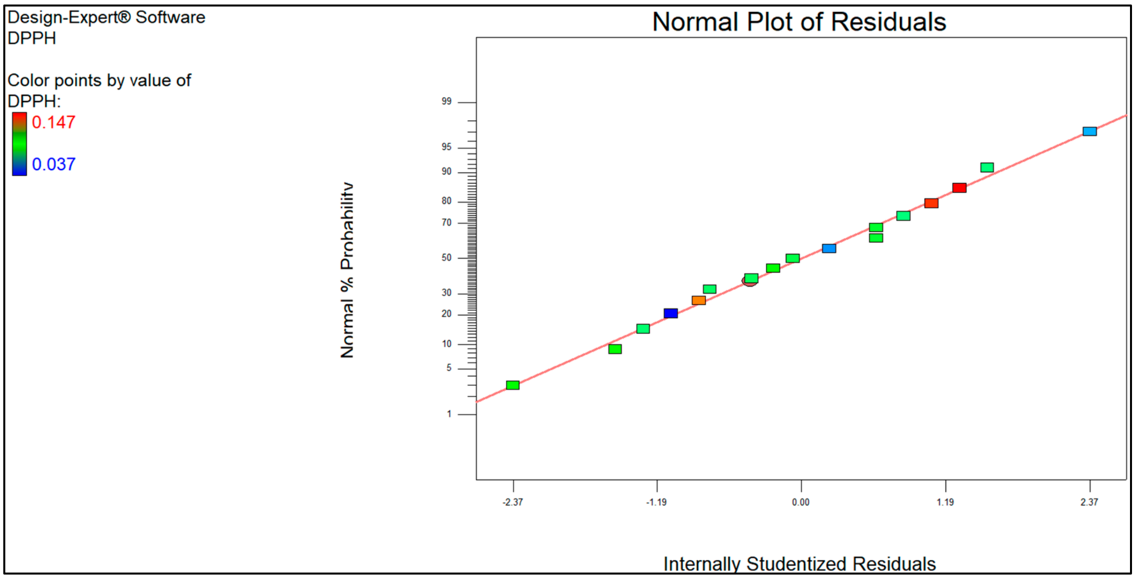
Design-Expert® Software
DPPH
Color points by value of
DPPH:
0.147
0.037
Normal Plot of Residuals
Normal % Probability
99
95
90
80
70
50
30
20
10
5
1
-2.37
-1.19
0.00
1.19
2.37
Internally Studentized Residuals

(d)

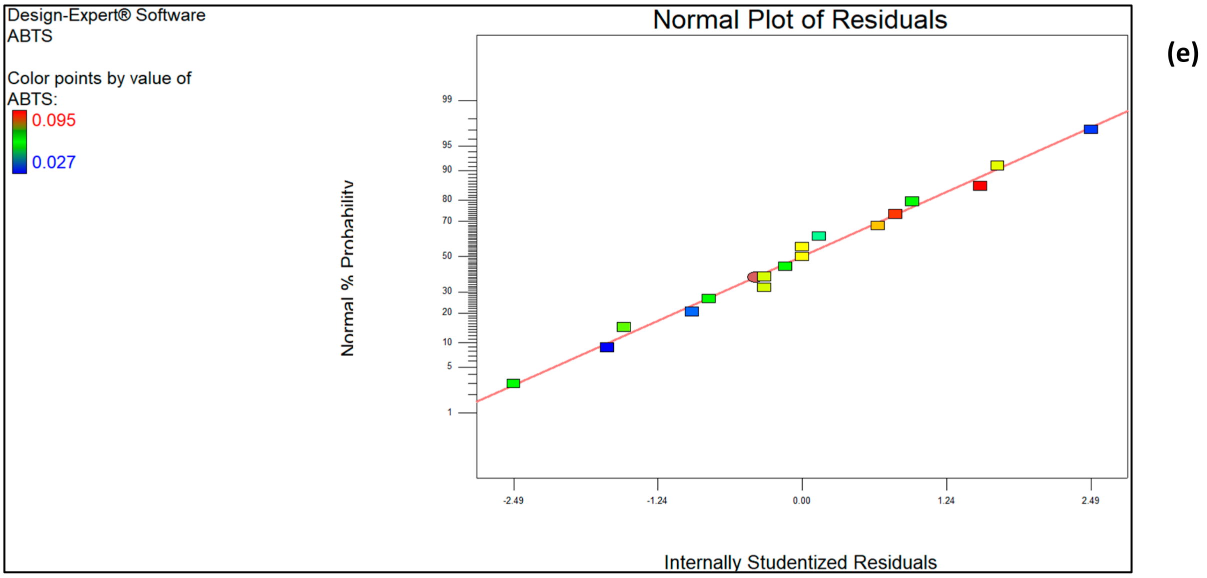

Figure S3 Normal plots of residuals for Carotenoids (a), Flavonoids (b), Polyphenols (c) DPPH (d) and ABTS (e) response. The near-linear distribution of points indicates normality of residuals

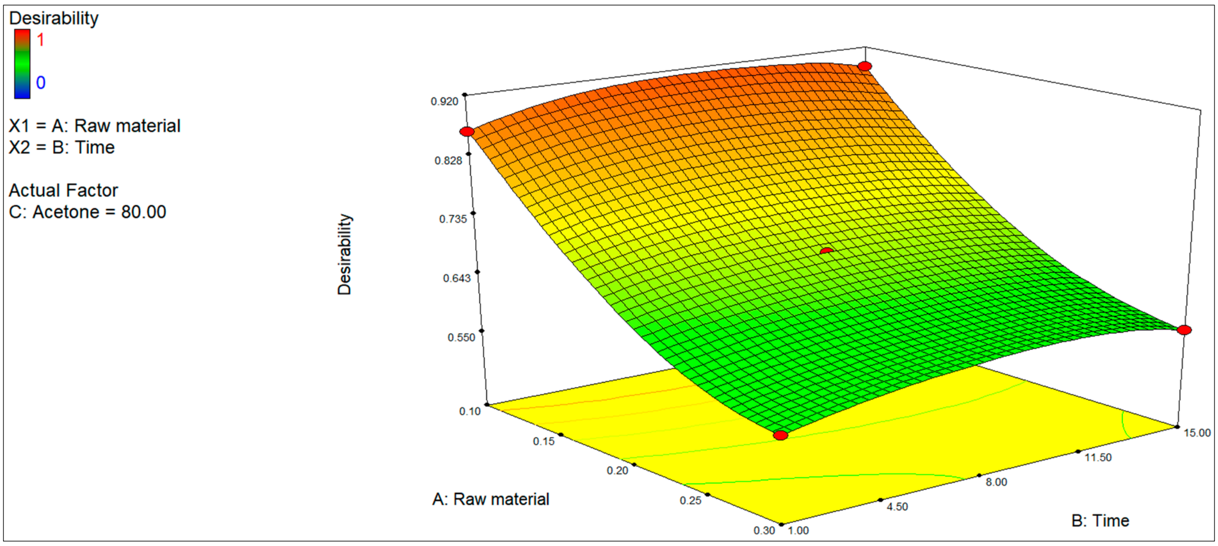

Figure S4 Response surface plot of overall desirability showing the combined effects of raw material concentration (A) and extraction time (B) at a fixed acetone concentration (80%). Maximum desirability (0.92) was achieved at 0.1% raw material, 8.97 min extraction time, and 80.06% (v/v) acetone.

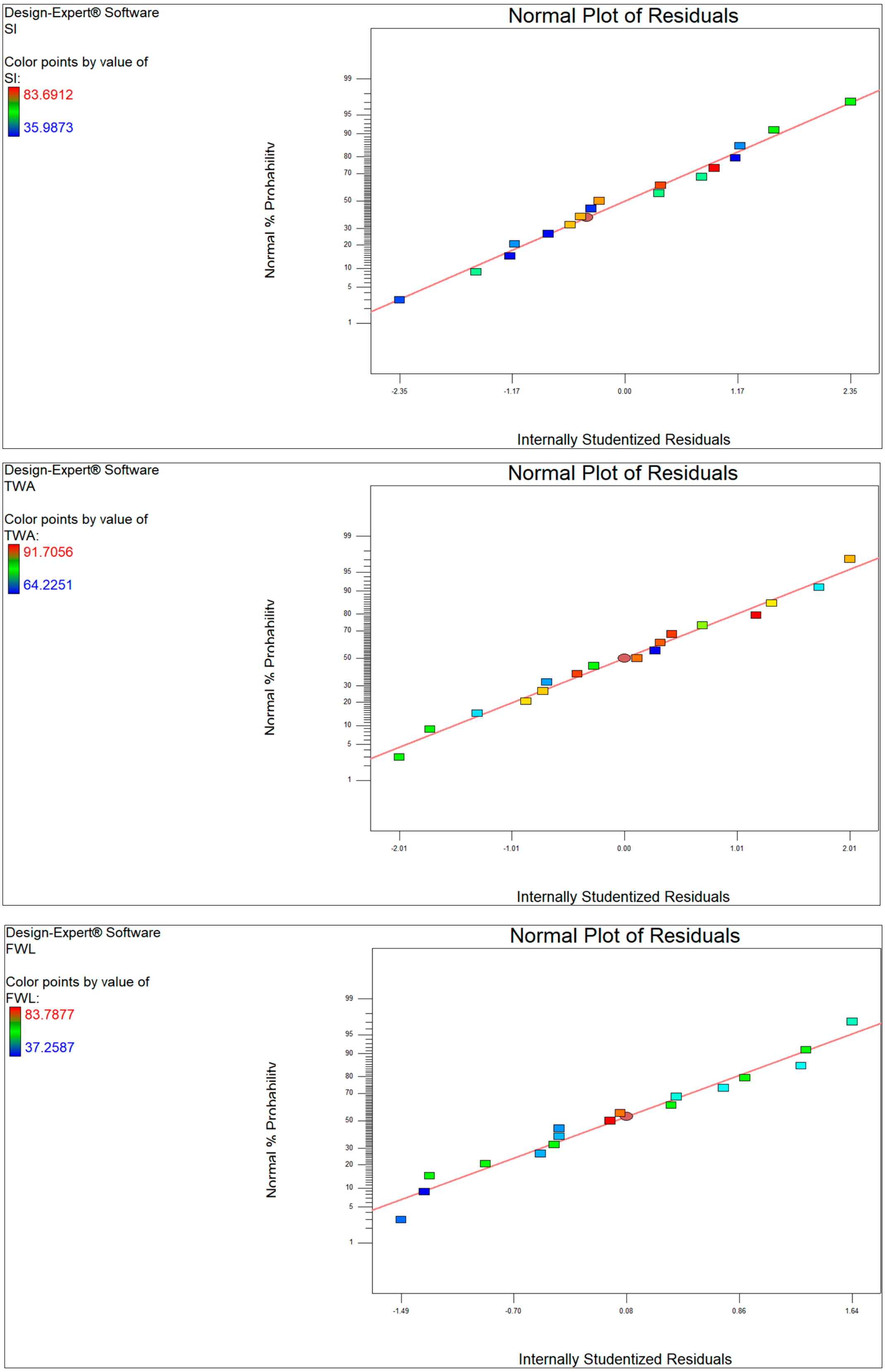

(a)

(b)

(c)

Figure S5 Normal plots of residues residuals for Swelling index (a), Total Water absorption (b) and Film Weigt loss (c) response. The near-linear distribution of points indicates normality of residuals

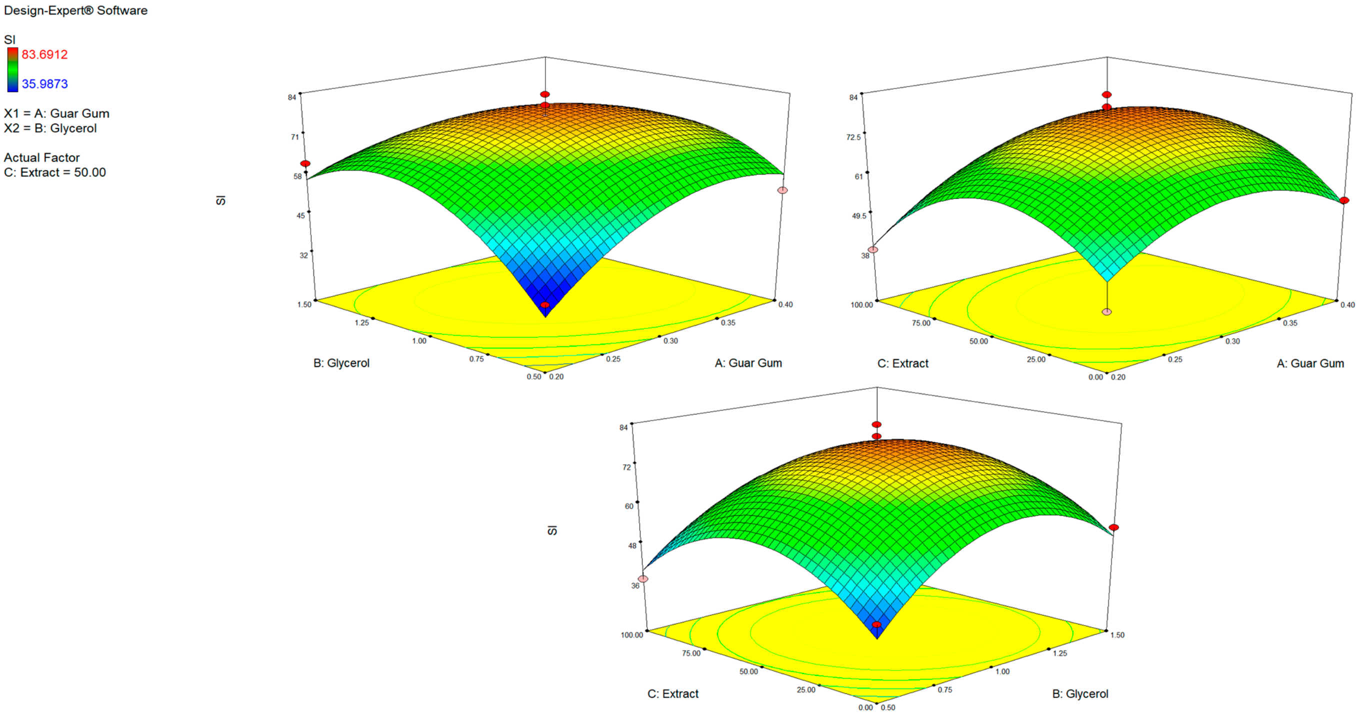

Figure S6 3D plots of Swelling Index (SI) showing the interactive effects of Guar gum/chitosan ratio (A) , Glycerol (B) and MPE extract (C)

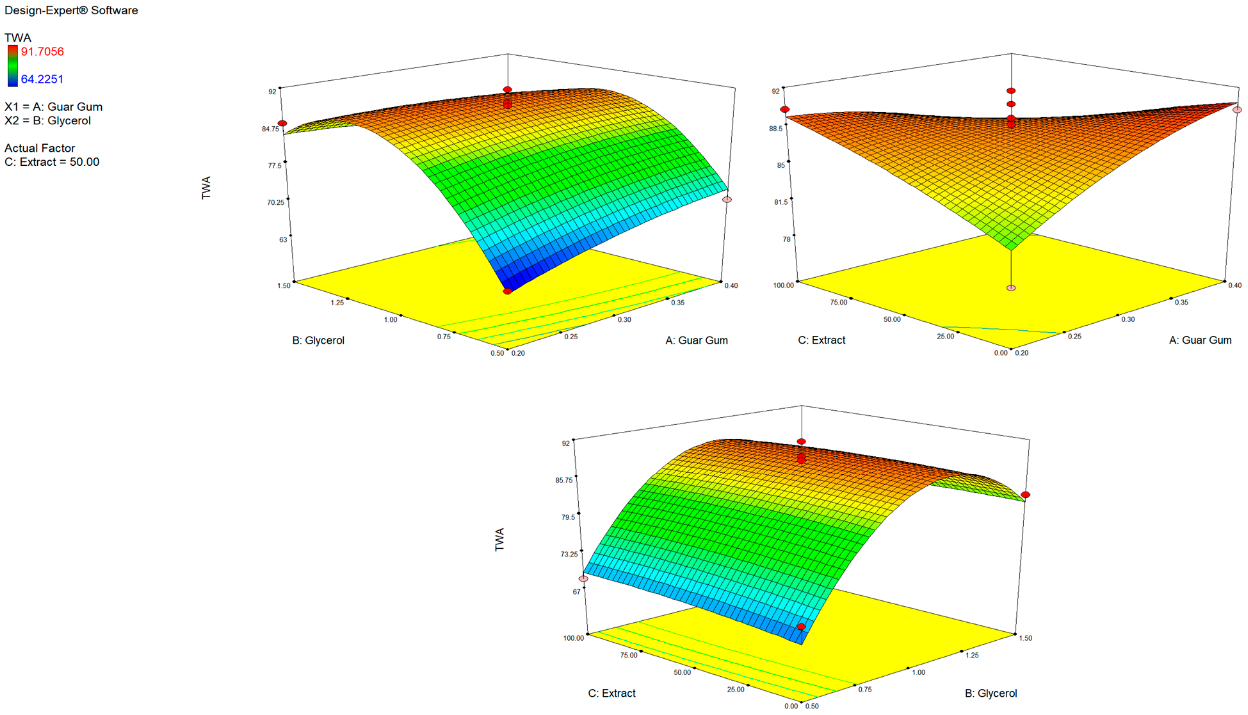

Figure S7 3D plots of Total Water Absorbation (TWA) showing the interactive effects of Guar gum/chitosan ratio (A) , Glycerol (B) and MPE extract (C)

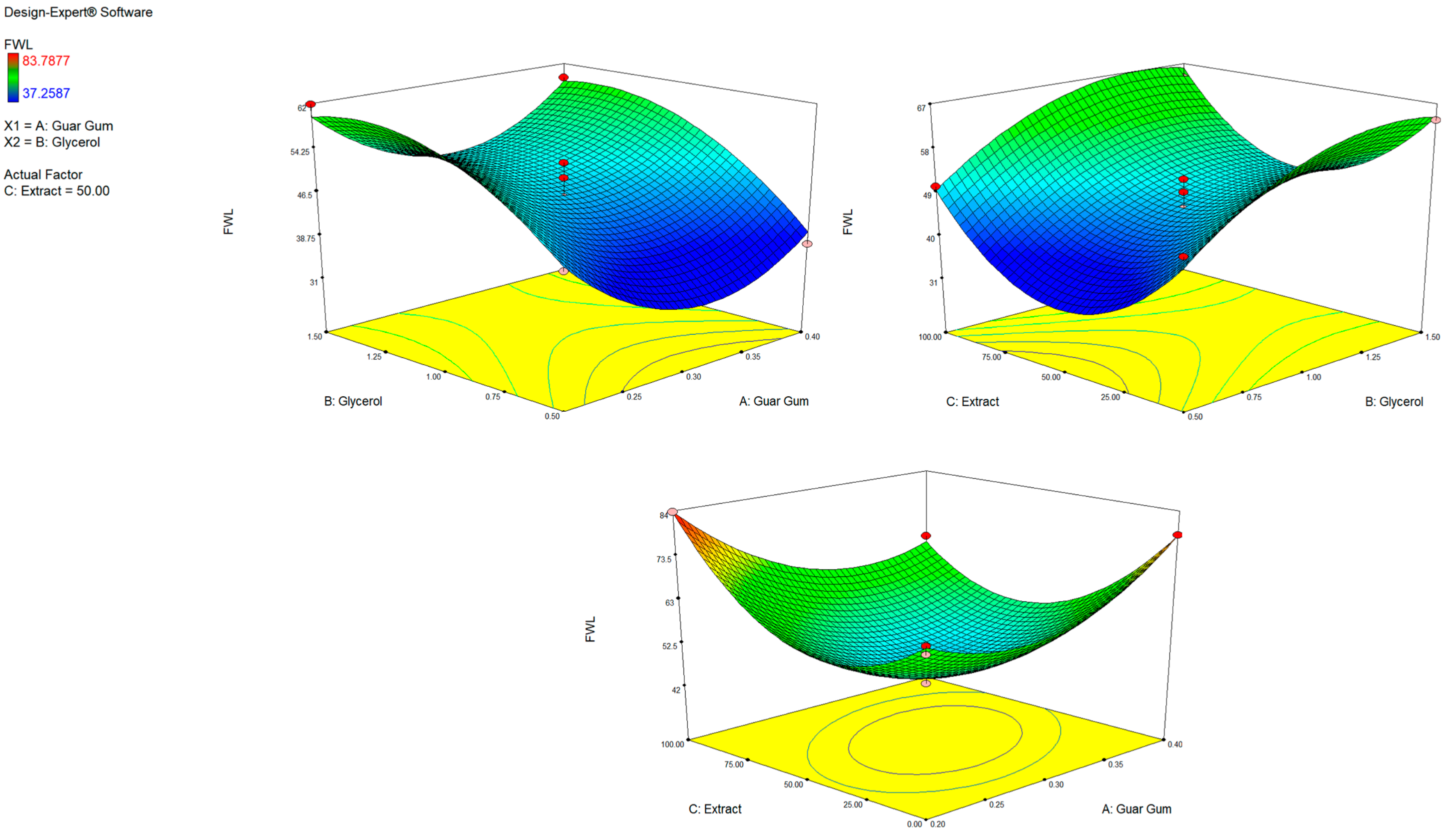

Figure S8 3D plots of Film Weight Loss (FWL) showing the interactive effects of Guar gum/chitosan ratio (A) , Glycerol (B) and MPE extract (C)

Table S1. Experimental design to optimize the mandarin peel extraction

Run	*F1*<br>*Raw material (%)*	*F2*<br>*Time (min)*	*F3*<br>*Acetone (%)*
1	0.10 (1 g/L)	15.00	80.00
2	0.20 (2 g/L)	8.00	80.00
3	0.30 (3 g/L)	8.00	60.00
4	0.20 (2 g/L)	8.00	80.00
5	0.10 (1 g/L)	8.00	100.00
6	0.20 (2 g/L)	15.00	60.00
7	0.20 (2 g/L)	1.00	60.00
8	0.30 (3 g/L)	15.00	80.00
9	0.30 (3 g/L)	8.00	100.00
10	0.10 (1 g/L)	8.00	60.00
11	0.20 (2 g/L)	1.00	100.00
12	0.20 (2 g/L)	8.00	80.00
13	0.20 (2 g/L)	8.00	80.00
14	0.30 (3 g/L)	1.00	80.00
15	0.20 (2 g/L)	8.00	80.00
16	0.10 (1 g/L)	1.00	80.00
17	0.20 (2 g/L)	15.00	100.00
